# Synthesis, Molecular Docking, and In Vitro Boron Neutron Capture Therapy Assay of Carboranyl Sinomenine †

**DOI:** 10.3390/molecules25204697

**Published:** 2020-10-14

**Authors:** Jianghong Cai, Narayan S. Hosmane, Masao Takagaki, Yinghuai Zhu

**Affiliations:** 1 School of Pharmacy, Macau University of Science and Technology, Avenida Wai Long, Taipa, Macau 999078, China; jhcai2020@sina.com; 2 Department of Chemistry and Biochemistry, Northern Illinois University, DeKalb, IL 60115, USA; hosmane@niu.edu; 3 Graduate School of Human and Environmental Studies, Kyoto University, Kyoto 604-8232, Japan; takagaki.masao.67x@st.kyoto-u.ac.jp

**Keywords:** boron neutron capture synovectomy (BNCS), boron neutron capture therapy (BNCT), anti-rheumatoid arthritis, carboranyl sinomenine, molecular docking

## Abstract

In comparison with pristine sinomenine and carborane precursors, the calculations of molecular docking with matrix metalloproteinases (MMPs) and methylcarboranyl-*n*-butyl sinomenine showed improved interactions. Accordingly, methylcarboranyl-*n*-butyl sinomenine shows a high potential in the treatment of rheumatoid arthritis (RA) in the presence of slow neutrons. The reaction of potassium salt of sinomenie, which is generated from the deprotonation of sinomenine (**1**) using potassium carbonate in a solvent of *N*,*N*-dimethyl formamide, with 4-methylcarboranyl-*n*-butyl iodide, (**2**) forms methylcarboranyl-*n*-butyl sinomenine (**3**) in 54.3% yield as a new product. This new compound was characterized by ^1^H, ^13^C, and ^11^B NMR spectroscopy, FT-IR spectroscopy, and elemental analyses to confirm its molecular composition. In addition to molecular docking interactions with MMPs, the in vitro killing effects of **3**, along with its toxicity measurements, exhibited its potential to be the new drug delivery agent for boron neutron capture synovectomy (BNCS) and boron neutron capture therapy (BNCT) for the treatment of rheumatoid arthritis (RA) and cancers in the presence of slow neutrons, respectively.

## 1. Introduction

Sinomenine, a naturally occurring bioactive alkaloid, is extracted from the root of the climbing plant *Sinomenium acutum* [1,2,3]. As one of the traditional Chinese medicines, sinomenine and its derived pharmaceutical products have been extensively developed and popularly used in the clinic to effectively relieve symptoms of rheumatoid arthritis (RA) and stop inflammation. Besides treating RA, sinomenine has also been used in other therapeutic drugs, such as drugs for heart failure (HF) and dysrhythmias as key components [4]. The RA is a chronic and destructive inflammatory disorder, affecting approximately 1% of the world’s population, and it is still growing [5]. In pharmacology, the RA usually affects the lining of the joints and shows inflammation with painful swelling, thickens the synovium, and eventually destroys the cartilage and erodes bone within the joint [6,7,8]. To date, there is no cure for RA clinically; all treatment medications, such as nonsteroidal anti-inflammatory drugs (NSAIDs), steroids, disease-modifying anti-rheumatic drugs (DMARDs), and biologic agents, are used to relieve symptoms and/or slow the progress of RA [6,7,8,9]. Therefore, it is highly desired to discover more effective drugs or technologies for RA treatment. In this regard, we are exploring the application of boron neutron capture therapy (BNCT) in RA treatment.

In theory, BNCT is a binary cancer treatment based on a nuclear fission reaction of a boron isotope (^10^B + ^1^n → [^11^B]* → ^4^He^2+^ (α) + ^7^Li^3+^ + 2.31 MeV). In the treatment, non-radioactive ^10^B atoms are selectively concentrated in a tumor target followed by thermal neutron irradiation to initiate the ^10^B nuclear fission reaction. The fission reaction releases high linear energy transfer (LET) α-particle (^4^He^2+^) and a ^7^Li^3+^ ion. These particles provide high energy along their very brief pathway (<10 μm), and, hence, their energy deposition is limited to the diameter of a single cell. Thus, only neoplastic cells with ^10^B are ravaged following thermal neutron irradiation [10,11]. The BNCT protocol is primarily focusing on the treatment of malignant brain tumors which tend to grow and aggressively infiltrate into the normal brain tissues so that surgeons are rarely able to remove all the cancerous tissues; therefore, if these tumors could be eradicated from the brain, there should be a significant increase in life expectancy [12,13,14,15]. In addition, malignant brain tumors are resistant to standard radiation treatment and chemotherapy and that will complicate the treatment [16]. Only two compounds, L-boronophenylalanine (L-BPA) and sodium borocaptate (BSH, Na_2_B_12_H_11_SH), have been approved by FDA and they are currently used for BNCT in clinic trials. Each compound uses a different mechanism to enter the malignant cells. The BSH passively diffuses from blood stream into malignant cells in the brain because these cells disrupt the blood–brain barrier (BBB). Meanwhile, the BPA is a boron-labelled amino acid which can be transported through cell membranes to the tumor at much higher rates in comparison with the healthy cells. The BPA concentration in malignant cells is around 2–4 times higher than in blood and other healthy cells. Unfortunately, neither BSH nor BPA show perfect selectivity and the uniform distribution in malignant cells. Thus, the results are not promising with these compounds, and hence there is a dire need for potential boron-10 carrier molecules and more sophisticated technologies that can selectively and homogeneously destroy tumor cells leading to the development of BNCT. Therefore, various boron agents such as porphyrins, liposomes, and antibodies, have been explored for selective delivery of boron atoms into tumor cells [17,18,19,20,21,22,23,24,25]. Nevertheless, only BPA and BSH are available for clinic use as of today and none of the new boron agents have been approved by FDA. Therefore, more research efforts are expected in developing highly efficient therapeutic boron agents.

On the other hand, in RA histopathology, the synovium is well recognized as the principal target [26]. Thus, surgical synovectomy has been used in RA treatment to reduce the acute inflammation of synovial tissues. Unfortunately, the RA symptoms typically reappear three years or so afterwards. Furthermore, surgical synovectomy may also cause complications of infection, neuro-algodystrophy, partial nervous palsy, and phlebitis. Therefore, more precise, less invasive and safer techniques are expected to remove acute inflammatory synovial tissues in RA. The BNCT has a high potential in RA treatment as an effective alternative procedure to synovectomy [27], since it selectively ravages tumor cells and spares the normal cells. Consequently, the boron neutron capture synovectomy (BNCS) aims to ablate the synovial membrane of RA joints. Clinically, the BNCS could be easily employed by following the two steps and they are: (**1**) injection of a boronated compound directly into the joint space, and (**2**) irradiation of the joint with the low energy thermal neutron beam to kill the inflammatory tissues after the boron compounds being taken up by the synovium. Since the boron agent is non-radioactive except during neutron irradiation, the BNCS radiation synovectomy is safe and easy to handle.

**Scheme 1:**
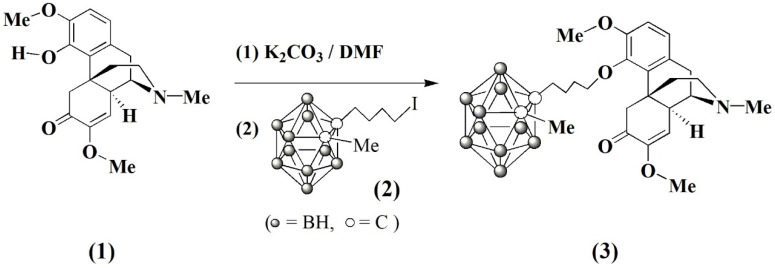
Synthesis of carboranyl sinomenine, 3.

Structurally, sinomenine (compound **1** in Scheme 1) is a morphinan derivative with a phenanthrene core skeleton with an aromatic ring, two saturated rings, and a nitrogen-containing six-membered saturated ring. This complicated molecular structure makes it a great challenge to totally synthesize and modify its molecular skeleton. Nevertheless, sinomenine can be mass-produced by a solvent-extraction technology in industry [28,29]. Therefore, application of sinomenine as one of the starting materials to develop the boron delivery agents is feasible due to its accountable industrial resources. In this work, the traditional Chinese medicine sinomenine has been functionalized with carboranyl cluster and used as a boron carrier molecule.

In medicinal chemistry, it is well recognized that the interaction between ligand and receptor plays crucial roles in whole pharmaceutical processes. These molecular associations may reveal the interaction mechanism of a drug and its target, and thus guides new drug development reversely. Following the advancement in the computer-assisted drug design, it has been realized to extensively elucidate the binding sites and various pathways occurred in a living cell by using molecular docking tools [30,31,32,33,34]. To asses our designing molecule of carboranyl sinomenine, compounds sinomenine (**1**), methyl-o-carborane, precursor of carborane moiety (**2**), and methylcarboranyl-*n*-butyl sinomenine (**3**) have been examined by molecular docking techniques. The matrix metalloproteinases (MMPs) such as interstitial collagenases MMP-1 and MMP-13 are enzymes that cleave protein substrates [35]. The MMP-1 and MMP-13 are responsible for the progression of both rheumatoid arthritis and osteoarthritis by degrading type II collagen in a cartilage [36]. It has been found that the product (**3**) always shows a higher score in comparison with pristine sinomenine and methyl carborane precursors. Herein, we report the synthesis, characterization, in vitro anti-RA and anti-tumor activities, and docking results.

## 2. Results and Discussion

### 2.1. Chemistry

Carboranyl sinomenine product was synthesized by a straightforward method as shown in Scheme 1. In this process, sinomenine anion was generated in situ from the reaction of sinomenine (**1**) with the excess quantity of potassium carbonate in *N*,*N*-dimethyl formide (DMF) solvent at room temperature under argon atmosphere. The resulting sinomenine anion reacted with 4-(methyl-*ortho*-carboranyl)-*n*-butyl iodide (**2**), prepared according to the literature method [17], to produce the carboranyl sinomenine (**3**) as a colorless solid in 54.3% yield after recrystallization from tetrahydrofuran (THF) solvent. The solid product 3 is soluble in various organic solvents, such as DMF, THF, and dimethyl sulfoxide (DMSO), etc.

The compound **3** was characterized by elemental analysis, NMR spectra and FT-IR spectra. All data are consistent with the formulations shown in Scheme 1. The ^1^H and ^13^C NMR spectra of **3** show normal resonances of methyl, methylene, and aromatic protons in the carboranyl cluster, along with -C_4_H_8_- bridge and sinomenine functional groups. In the ^11^B NMR {^1^H} spectra of **3**, four peaks (area ratio 1:1:2:1) at the chemical shifts (δ) of −2.43 (2B), −9.19 (2B), −13.67 (4B) and −14.74 (2B) ppm were observed, respectively, for the ^11^B resonances of BH groups of the carborane cage. In FT-IR spectra, the B-H bond stretching absorption (ν_B-H_) was found at 2594 cm^−1^ for **3**. The C=O bond stretching absorptions were observed at around 1680 cm^−1^. The chemical analysis was carried out to further characterize the new products, and the results were consistent to support their molecular structures.

### 2.2. Molecular Docking

**Figure 1:**
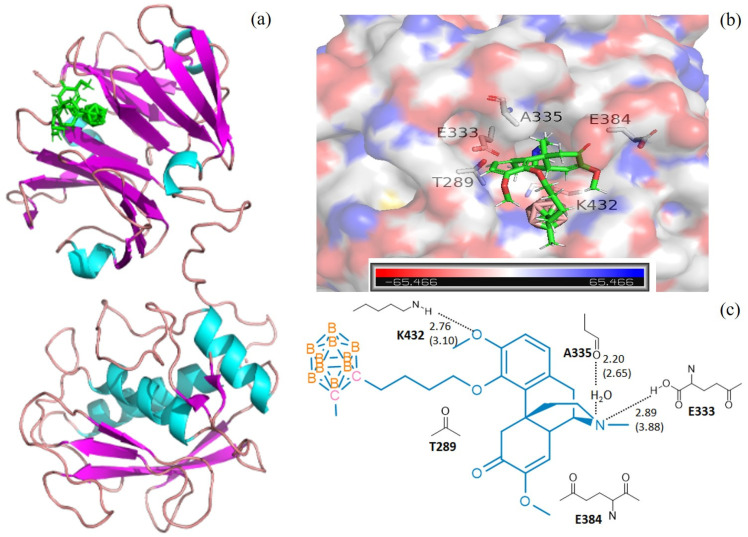
The docking models for **3** with collagenases MMP1-1FBL. (**a**) domain structures of the interaction of **3** (green) and MMP1-1FBL (ribbon diagram illustrating). The figure was generated using the pymol 2.4 software; (**b**) MMP1-1FBL active site pocket with bound moiety of **3**. The collagenase surface is colored by electrostatic potential; blue for positive and red for negative. The figure was generated using the pymol 2.4 software; (**c**) interactions (hydrogen bonds) of amino acid residues with **3**. The figure was generated using Chemdraw 10.0 and pymol 2.4 software.

**Figure 2:**
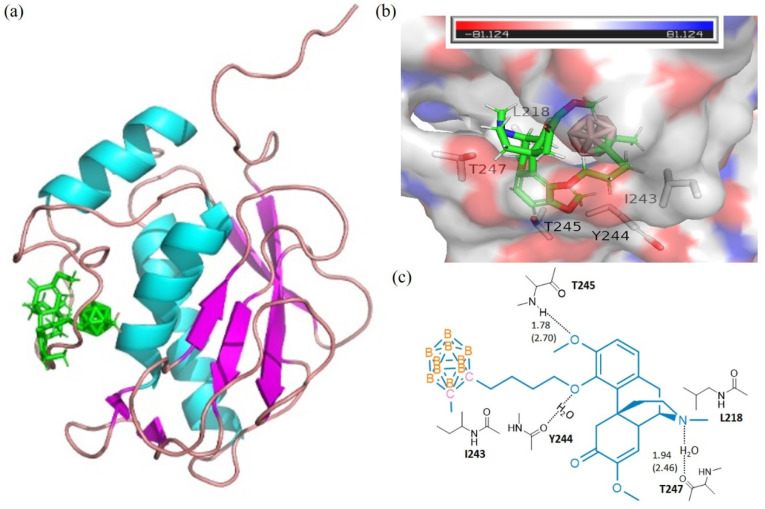
The docking models for **3** with collagenases MMP13-2YIG. (**a**) domain structures of the interaction of **3** (green) and MMP13-2YIG (ribbon diagram illustrating). The figure was generated using the pymol 2.4 software. (**b**) MMP13-2YIG active site pocket with bound moiety of **3**. The collagenase surface is colored by electrostatic potential; blue for positive and red for negative. The figure was generated using the pymol 2.4 software; (**c**) interactions (hydrogen bonds) of amino acid residues with **3**. The figure was generated using Chemdraw 10.0 and pymol 2.4 software.

The calculations of molecular docking algorithm based on shaped complementarity principle are listed in Supplementary Table S1. It has been observed that the title compound **3** consistently demonstrated higher geometric shape complementarity score when compared to that of pristine sinomenine and carborane precursors for examining MMP-1 and MMP-13 interstitial collagenases. Figure 1 and Figure 2 show the highly probable and energetically favorable docked models of **3** with MMP-1 (collagenase 1FBL) and MMP-13 (collagenase 2YIG), respectively. The domain structures of direct active regional residues with **3** (green) are illustrated in Figure 1a and Figure 2a. In addition, Figure 1a and Figure 2a represent the active site pockets of MMPs bound with the ligand in **3**, which were generated by Pymol 2.4 and Chemdraw 10.0 software packages. Both the oxygen atoms attaching to the benzene ring and nitrogen atoms in the sinomenine species form hydrogen bonds to lysine 432, glutamic acid 333, and threonine 254, respectively. The icosahedral carbaborane moiety is extremely hydrophobic [37], which may increase the receptor binding with the hydrophobic amino acids in the docking pocket. In addition, the BH units in the cluster are capable of forming dihydrogen bonds (B−H⋅⋅⋅H−X) (X = O, N, S, F, π system). According to Figure 1c and Figure 2c, there are hydrophobic amino acids, such as alanine (A) 335, isoleucine (I) 243, isoleucine (I) 218, and polar amino acids, such as threonine (T) 289, threonine (T) 245, threonine (T) 247, lysine (K) 432, glutamic acid (E) 333, glutamic acid (E) 384, and tyrosine (Y) 244. Therefore, there are reasonable Van der Waals interactions between the methyl carboranyl group of **3** with the lipophilic parts in the MMP-1 and MMP-13. Meanwhile, the dihydrogen bonds should also be created when **3** interacts with polar amino acids in the protein pocket of the MMP1 and MMP13, which warrants further investigations.

It has been found that **3** consistently demonstrated higher geometric shape complementarity (GSC) score in comparison with those of pristine precursors of sinmenine and 1-methyl-*closo*-1,2-dicarbaborane based on the calculations of molecular docking. While among investigating MMP-1 interstitial collagenases, the docked model of 1FBL (Figure 2) with the highest GSC score of 4536 for **3**: MMP1-1FBL was calculated. In comparison, sinomenine: MMP1-1FBL and 1-methyl-*closo*-1,2-dicarbaborane: MMP1-1FBL showed GSC score of 3970 and 2242, respectively. Similarly, the molecular docking of **3** with MMP-13 interstitial collagenases led us to calculate 49 collagenase receptors. For example, the **3**: MMP13-2YIG docking model showed the highest GSC score of 5116, while sinomenine: MMP13-2YIG and 1-methyl-*closo*-1,2-dicarbaborane: MMP13-2YIG docking showed the GSC score of 4648 and 2226, respectively. The results of molecular docking strongly suggested that the compound **3** effectively interacts with MMPs. In addition, we use Molecular Operating Environment (MOE) software (MOE 2016.08 software package, Chemical Computing Group ULC, Montreal, QC, Canada) to study the binding energy of MMP1-1FBL and MMP13-2YIG for compounds **1**–**3** by applying the AMBER10 force field [38]. The calculating results are listed at Table 1. For the collagenases of MMP1-1FBL and MMP13-2YIG, compound **3** shows the lowest binding energy of –7.7927 and –8.5128 kcal/mol, respectively, in comparison with the binding energy of compounds **1** and **2**. The docking calculation of the binding energy from MOE is consistent with the GSC results obtained from the PatchDock source. Therefore, the title compound **3** is reasonably expected to offer potential promising in vivo anti-RA activity.

### 2.3. Bioassessment

**Figure 3:**
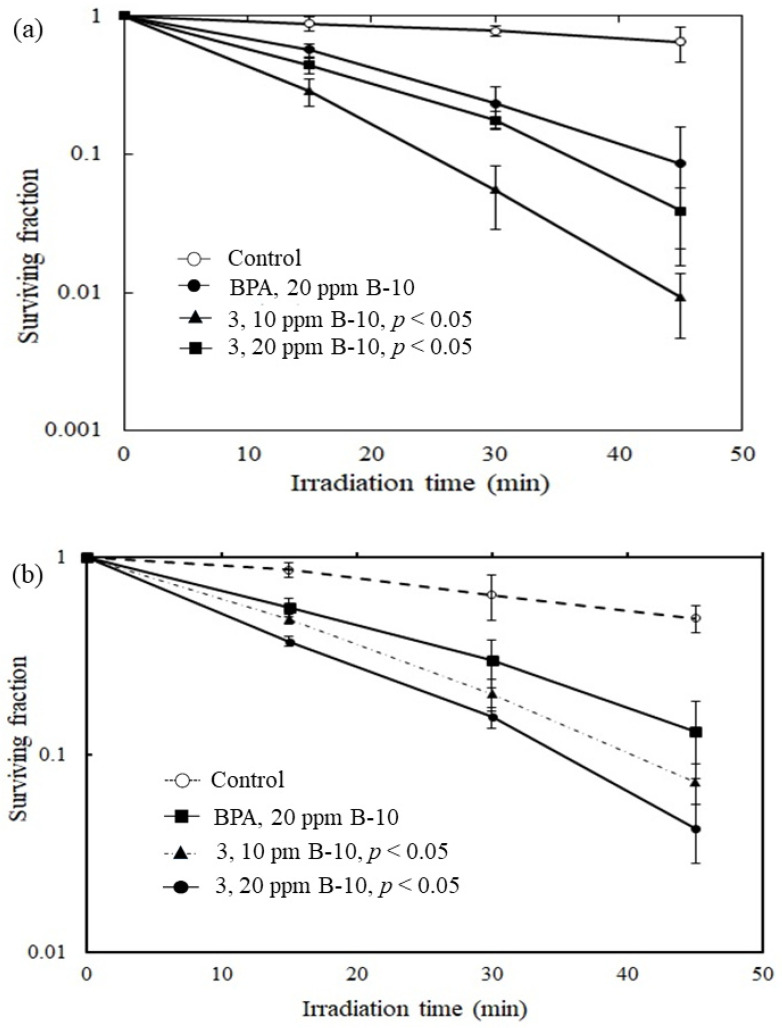
Plots of surviving fraction vs. irradiation time. (**a**) The C6 gliosarcoma cells. It shows the preliminary in vitro killing effects of **3** where it has been observed that **3** demonstrated significantly improved killing effects for C6 gliosarcoma tumor cells when compared to that with the clinical drug BPA under the same operating conditions. (**b**) The rat fibroblast-like synoviocytes (FLS) cells, compound **3** shows similar improved killing effects when compared to that with BPA. *p* < 0.05 relative to control.

The preliminary in vitro killing effects of compound **3** were examined by using rat C6 gliosarcoma tumor cells and fibroblast-like synoviocytes (FLS) cells, respectively. The in vitro survival study was completed with the thermal neutron fluence ranging from 0 to 4.2 × 10^12^ n/cm^2^. The γ-ray dose presented in the experiment was monitored by thermoluminescent dosimeters (TLDs) attached to the tubes and ranged from 0 to 5 Sv. As shown in Figure 3a, compound **3** demonstrated significantly improved killing effects for C6 gliosarcoma tumor cells. A low surviving fraction of around 0.01 was obtained by compound **3** after irradiation for 50 min. Compared with the current clinically used BNCT drug 4-borono-L-phenylalanine (L-BPA), the killing effect has been improved approximately nine times under the same operating conditions. As shown in Figure 3b, compound **3** demonstrated significantly improved killing effects for FLS cells. Compared with the current clinically used (L-BPA), the killing effects has been enhanced approximately three times under the same operating conditions. In a cytotoxicity study, an in vitro IC_50_ value of 310.4 ± 0.3 μM for **3** was obtained according to the dose responsive curve using rat FLS cells (Supplementary Figure S5).

The rat C6 glioma cells and fibroblast-like synoviocytes (FLS) cells were used to conduct the boron uptake of **3**. The fractional amounts delivered to these cells are summarized in Table 2. The boron uptakes of clinically used L-BPA and BSH were also listed for comparation. The results reveal that **3** was able to permeate both C6 glioma cells and FLS cells. Since the in vitro boron uptakes of **3** were significantly higher than that of L-BPA and BSH, the results also demonstrated that **3** was more permeable towards the examined cell membranes. The higher membrane permeability provides enhanced boron concentration in the cell lines. This could partially support that **3** showed a high capacity in vitro cell killing effects. Nevertheless, the detailed mechanism of **3** crossing cell membrane warrants further investigation.

## 3. Materials and Methods

### 3.1. Materials and Equipment

All reactions were carried out under an argon atmosphere using standard Schlenk-line techniques. Tetrahydrofuran (THF) and diethyl ether were dried over sodium and freshly distilled before use. The 4-(methyl-*ortho*-carboranyl)-*n*-butyl iodide was synthesized according to the literature procedure [17]. The 1-methyl-*ortho*-carborane, 1,4-diiodobutane, sinomenine, potassium carbonate and other reagents and organic solvents, were used as received from Sigma-Aldrich (Singapore). The FT-IR spectra were measured using an IRTracer-100 Shimadzu spectrophotometer with KBr pellets (Macau, China). The FT-IR multiplicities are reported as (peak shape, strength): s = singlet, vs = very strong, m = medium, w = weak. Elemental analyses were measured using a EURO EA equipment (Singapore). High-resolution mass spectra were obtained using Waters Q-TOF Ultima ESI (Singapore) and Agilent 6230 ESI TOF LC/MS spectrometers (Singapore). The ^1^H, ^13^C, and ^11^B NMR spectra were recorded using a Bruker 200 analyzer (Singapore) at 200, 64.2 and 50.3 MHz, respectively. ^1^H NMR multiplicities are reported as: s = singlet, d = doublet, t = triplet, q = quartet, m = multiplet, br = broad. All NMR spectra were recorded at ambient temperature. Inductively coupled plasma-optical emission spectroscopy (ICP-OES) measurements were carried out using a VISTA-MPX machine (Singapore).

### 3.2. Synthesis of **3**

A 0.330 g (0.1 mmol) sample of sinomenine was added to a three-necked round-bottom flask equipped with a stirring bar, a solid additional tube containing a 0.415 g (3.0 mmol) fine powder of potassium carbonate, and an argon inlet. The sinomenine solid was dissolved in 30.0 mL N, N-dimethylformamide, resulting a transparent colorless solution, which was cooled to −10 °C with a cooling bath. Then, the potassium carbonate was carefully added in portions to maintain the temperature. After addition, the mixture was stirring at the same temperature for 1 h before warming to 0 °C spontaneously. The reaction was continued for additional 5 h and filtered under argon to give a transparent solution. The transparent solution was added dropwise to a solution containing 0.374 g (0.11 mmol) of 1-(methyl-*ortho*-carboranyl)-*n*-butyl iodide in 10.0 mL of *N*,*N*-dimethylformamide at −10 °C with vigorous stirring. After complete addition, the resulting mixture was stirring at the same temperature for 1 h before warming to 0 °C, spontaneously. The reaction was continued for additional 3 h at 0 °C followed by another 4 h at room temperature. The resulting reaction mixture was then filtered under argon to collect a pale-yellow transparent solution. All volatiles were removed from this solution under reduced pressure to obtain an off-white solid residue which was further subjected to recrystallization in a solvent mixture of petroleum ether (30–60 °C) and tetrahydrofuran to produce 0.294 g off-white solid of (**3**) (m.p. 114–116 °C) in 54.3% yield. Elemental Anal: Calcd for C_26_H_43_B_10_NO_4_: C, 57.65; H, 8.00; N, 2.59; B, 19.96. Found: C, 57.64; H, 8.03; N, 2.58; B, 19.94 (ICP-MS analysis). HRMS(ESI): *m*/*z* calc. for C_26_H_43_B_10_NO_4_ [M + H]^+^: 544.4201 found: 544.4200. ^1^H NMR (DMSO-d_6_, relative to SiMe_4_; ppm): δ 6.62–5.84 (m, 2H, C_6_H_2_), 5.72 (d, 1H, C(OMe)=CH-), 4.53 (t, 2H, -OCH_2_-), 4.21 (s, 3H, Ar-OCH_3_), 4.12 (s, 3H, =C-OCH_3_), 3.31 (m, 1H, CO-CH), 3.14, 3.09 (m, 2H, CO-CH, Ar-CH), 2.92–1,10 (m, 29H, 5CH_2_, Ar-CH, N-CH_3_, N-CH-C, -HC(-C)-C=, C_Cage_-CH_3_, C_2_B_10_H_10_). ^13^C NMR (DMSO-d_6_, relative to SiMe_4_; ppm): δ 192.2 (1C, CO), 150.9, 144.7, 144.3, 130.0, 122.7, 117.1, 115.8, 109.0, 78.6 (1C, C_Cage_), 75.6 (1C, C_Cage_), 62.4, 55.2, 55.1, 54.8, 47.9, 46.1, 45.9, 41.8, 39.3, 34.6, 33.0, 31.5, 29.9, 23.3, 21.9. ^11^B NMR (DMSO-d_6_, relative to BF_3_⋅OEt_2_, ^1^H-decoupled; ppm): δ −2.43 (2B), −9.19 (2B), −13.67 (4B), 14.74 (2B). IR (film on KBr, cm^−1^): 3446.8 (br, vs), 2937.6 (s, s), 2594.3 (s, vs, ν_BH_), 1680.0 (s, vs), 1654.9 (s, s), 1624.1 (s, s), 1560.4 (s, m), 1489.1 (s, m), 1437.0 (s, m), 1379.1 (s, w), 1278.8 (s, s), 1232.5 (s, w), 1203.6 (s, m), 1147.7 (s, m), 1091.7 (s, w), 1055.1 (s, m), 885.3 (s, w), 808.2 (s, w), 738.7 (s, w), 636.5 (s, w).

### 3.3. Molecular Docking Calculation

The molecular operating environment (MOE) software (Chemical Computing Group 2016, Montreal, Canada) and protein-small molecule docking study Patch dock open source server (http://bioinfo3d.cs.tau.ac.il/PatchDock/) [30,31,32,33], which can predict favorable protein–ligand complex structures with high accuracy have been used to calculate the molecular interactions between ligand (small molecule) and receptor (protein). The three-dimensional (3D) crystal structures of MMP1 and MMP13 proteins were retrieved from the Protein Data Bank (PDB) (www.rcsb.org). Solvent impurities and co-crystallized ligand molecule and all water molecules were manually removed by the Pymol 2.4 Software package, and then saved the proteins as. pdb files after adding polar hydrogen atoms to the proteins. Both PDB files were uploaded into the Patch dock server for protein- small molecule docking simulation. Docking was performed with complex type configuration settings. The PatchDock server follows a geometry-based molecular docking algorithm to find the docking transformations with good molecular shape complementarity. The PatchDock algorithm separates the Connolly dot surface representation of the molecules into concave, convex, and flat patches. These divided complementary patches are matched in order to generate candidate transformations and evaluated by geometric fit and atomic desolvation energy scoring functions. The results show binding sites for **3**. The association details of each binding site are presented using the “ligand interactions” module of MOE. The calculated results of geometric shape complementarity score are listed at Supplementary Table S1. The docking models of **3** with varying MMPs are shown in Figure 1 and Figure 2.

The binding energy was calculated using a Molecular Operating Environment (MOE) software (MOE 2016.08 software package, Chemical Computing Group ULC, Montreal, QC, Canada). The software establishes a good correlation between the binding strength and various physico-chemical parameters. The coefficients were fitted from approximately 400 X-ray crystal structures of protein-ligand complexes with available experimental pKi data. Atoms are categorized into about a dozen atom types for the assignment of the ci coefficients. The triple integrals are approximated using Generalized Born integral formulas. The proteins were downloaded from online RCSB Protein Data Bank (PDB). Energy minimization was performed using defaults parameters in MOE by applying the AMBER10 force field [38]. The conformational searching of the compounds **1**–**3** was conducted by the LowModeMD method. The docking function of MOE can give the correct conformation of the compounds **1**–**3** to obtain minimum energy structure. Binding energy (S score) was the basis for the choice of top conformation for each compound. The top conformations also received further evaluation in order to study the hydrogen bonding/π–π interactions.

### 3.4. In Vitro Survival Study

The in vitro assessments were carried out according to methods reported in the literature [21,34]. In the procedure, the rat C6 gliosarcoma cells and the rat fibroblast-like synoviocytes (FLS) cells were incubated in subconfluent conditions in standard minimum essential medium (MEM) supplemented with 10% fetal calf serum (FCS), at 36 °C and 5% CO_2_ atmosphere for overnight. Aliquots of the boron sample solutions were added over a period of 6 h into the respective Petri dishes for uptaking of the rat C6 gliosarcoma cells in dose-dependent manners. The boron concentrations in the media were confirmed by prompt γ spectroscopy (PGA) (Kyoto, Japan). The C6 cells were washed twice after boron loading. Aliquots containing 6.0 × 10^3^ cells/mL were pipetted into cylindrical Teflon tubes of 1 cm in diameter and 3 cm high which did not generate any secondary radiation when subjected to thermal neutrons. The inability of cells to adhere to Teflon allows precise quantitative manipulation of cells without trypsinization. In the case of the rat FLS cells, an aliquot containing approximately 6.5 × 10^3^ cells/mL were pipetted into the similar cylindrical Teflon tubes for further analysis. The thermal neutron fluence was determined by averaging the activity of two gold films symmetrically attached to the Teflon tube surface along the thermal neutron axis. The thermal neutron fluence ranged from 0 to 4.2 × 10^12^ n/cm^2^. For estimation of the neutron energy spectra, 8 kinds of activation films and 14 kinds of nuclear reactions were used: ^197^Au(n, γ)^198^Au for the thermal neutron regions; ^115^In(n, γ)^116m^In, ^197^Au(n, γ)^198^Au, ^58^Fe(n, γ)^59^Fe and ^63^Cu(n, γ)^64^Cu for the epithermal neutron region; and ^115^In(n, n*)^116^In, ^54^Fe(n, p)^54^Mn, ^27^Al(n, p)^27^Mg, ^27^Al(n, α)^24^Na, ^47^Ti(n, p)^47^Sc, ^48^Ti(n, p)^48^Sc, ^24^Mg(n, p)^24^Na, ^63^Cu(n, α)^60^Co, ^58^Ni(n, p)^58^Co and ^197^Au(n, 2n)^196^Au for the fast-neutron region. The neutron absorbed dose (Gy) was calculated using the flux-to-dose conversion factor [39,40]. The elemental composition of the tumors was assumed to be 10.7% hydrogen, 12.1% carbon, 2% nitrogen, 71.4% oxygen, and 3.8% other elements [34,35]. The γ-ray dose was monitored by thermoluminescent dosimeters (TLDs) (Kyoto, Japan) attached to the tubes, and it ranged from 0 to 5 Sv. Immediately after irradiation, 500 cells were seeded in 6-cm Petri dishes (Corning, NY, USA) and incubated for 10 days in a humidified 37 °C atmosphere of 95% air / 5% carbon dioxide to allow colony formation. The colonies were fixed and stained with a 10% formaldehyde/1% toluidine blue solution and then counted microscopically.

### 3.5. Cytotoxicity Study

The IC_50_ (moles/liter, *M*), the concentration that inhibited the growth of rat FLS cells by 50% after 3 days of continuous exposure, was determined using the standard MTT assay. Suspensions of 4.9 × 10^5^ of rat fibroblast-like synoviocytes cells /200 μL MEM containing 20% FCS were incubated for 3 days using 96-well culture plates with various concentrations of the samples in dimethyl sulfoxide (DMSO). The damaged cells were detected at 490 nm optical absorbance by Aqueous One Solution Cell Proliferation Assay. The IC_50_ value of 310.4 ± 0.3 μM was obtained for **3**, and the dose responsive curve was shown in Supplementary Figure S1.

### 3.6. Uptake of Boron by Rat Fibroblast-Like Synoviocytes Cells and Rat C6 Gliosarcoma Cells

According to literature [21,31], 3.7 × 10^8^ rat FLS cells or 2.6 × 10^7^ rat C6 glioma cells were seeded and cultured at room temperature (37 °C, 5% CO) for 24 hrs. This culture fluid was removed therefrom by suction, culture fluids containing 1.5 mM of boron agent **3** with the boron concentrations of 3.0 mM, 1.5 mM, 18.0 mM, and 1.5 mM, respectively, were added and continued culturing for 24 h under the same conditions. These culture fluids were removed by suction and the cells were washed three times with PBS and treated with trypsin to recover the cells. The number of cells recovered was counted, and HNO_3_ (2N, 1.5 mL) was added and the resulting mixture was heated at 80 °C for 12 h. After filtering using a membrane filter, the boron concentration was determined by ICP-AES (Singapore).

### 3.7. Statistical Analysis

The in vitro BNCS was carried out in triplicate. Values are the mean ± SEM from three independent experiments. The significance of the differences in survival rates was assessed by Student’s *t*-test. The statistical analyses were performed using Prism 3.0 (GraphPad Software Inc., La Jolla, CA, USA).

## 4. Conclusions

In conclusion, the active species of traditional Chinese medicine, sinomenine, was conjugated with the carborane cluster for developing both the traditional Chinese medicine and BNCT. The molecular docking calculations revealed that the resulting compound **3** intensively interacted with MMPs, such as interstitial collagenases MMP-1 and MMP-13 in comparison with those of pristine precursors of sinomenine and methyl-*ortho*-carborane, respectively. Compound **3** demonstrated significantly enhanced in vitro cell killing capacity for both C6 tumor cells and FLS cells in compared to clinically used boron drug, BPA. Compound **3** also showed higher membrane permeability in the cell lines than those with L-BPA and BSH, and this is consistent with the results of in vitro cell killing effects. All of these results warrant further investigation of compound **3** as a potential and effective BNCT and BNCS agent. Further bioassessments and cytotoxicity studies on a similar class of boron compounds are currently underway in our laboratories.

## Figures and Tables

**Table 1:** Binding energy of compounds **1**–**3** (kcal/mol).

	Compound	1	2	3
MMP				
MMP1-1FBL		−6.8011	−5.3157	−7.7927
MMP13-2YIG		−7.0156	−6.6171	−8.5128

**Table 2:** Boron amounts delivered to the cells (μg/10^7^ cells).

	Compound	3	L-BPA	BSH
Cell Line				
C6 glioma cells		4.25	0.67 [21]	2.14 [21]
FLS cells		3.04	0.74 [31]	1.82 [31]

## Supplementary Materials

Supplementary Information shows the synthetic methods in general, cytotoxicity study and molecular docking original data.
