# Supplementary material for: Synthesis, Molecular Docking, and In Vitro Boron Neutron Capture Therapy Assay of Carboranyl Sinomenine
Source: Molecules. 2020 Oct 14;25(20):4697. doi: 10.3390/molecules25204697 (PMC7587333; doi:10.3390/molecules25204697)
Supplement: Supplementary file 1 [file molecules-25-04697-s001.pdf]

Synthesis, Molecular Docking and *in vitro* Boron Neutron Capture Therapy Assay of Carboranyl Sinomenine

Jianghong Cai [1], Narayan S Hosmane [2], Masao Takagaki [3], Yinghuai Zhu [1,*]

[1] School of Pharmacy, Macau University of Science and Technology, Avenida Wai Long, Taipa, Macau 999078, China (zhu_yinghuai@163.com);

[2] Department of Chemistry and Biochemistry, Northern Illinois University, DeKalb, Illinois 60115, USA;

[3] Graduate School of Human and Environmental Studies, Kyoto University, Kyoto 604-8232, Japan

Experimental Section:

1. General. All reactions were carried out under an argon atmosphere using standard Schlenk-line techniques. Tetrahydrofuran (THF) and diethyl ether were dried over sodium and freshly distilled before use. The 4-(methyl-*ortho*-carboranyl)-*n*-butyl iodide was synthesized according to the literature procedure [1]. The 1-methyl-*ortho*-carborane, 1,4-diiodobutane, sinomenine, potassium carbonate and other reagents and organic solvents, were used as received from Sigma-Aldrich. The FT-IR spectra were measured using an IRTracer-100 SHIMADZU spectrophotometer with KBr pellets. Elemental analyses were measured using a EURO EA equipment. High–resolution mass spectra were obtained using Waters Q-TOF Ultima ESI and Agilent 6230 ESI TOF LC/MS spectrometers. The ^{1}H, ^{13}C, and ^{11}B NMR spectra were recorded using a Bruker 200 analyzer at 200, 64.2 and 50.3 MHz, respectively. All NMR spectra were recorded at ambient temperature. Inductively coupled plasma-optical emission spectroscopy (ICP-OES) measurements were carried out using a VISTA-MPX machine.

Figure S-1. FT-IR spectra of **3**.

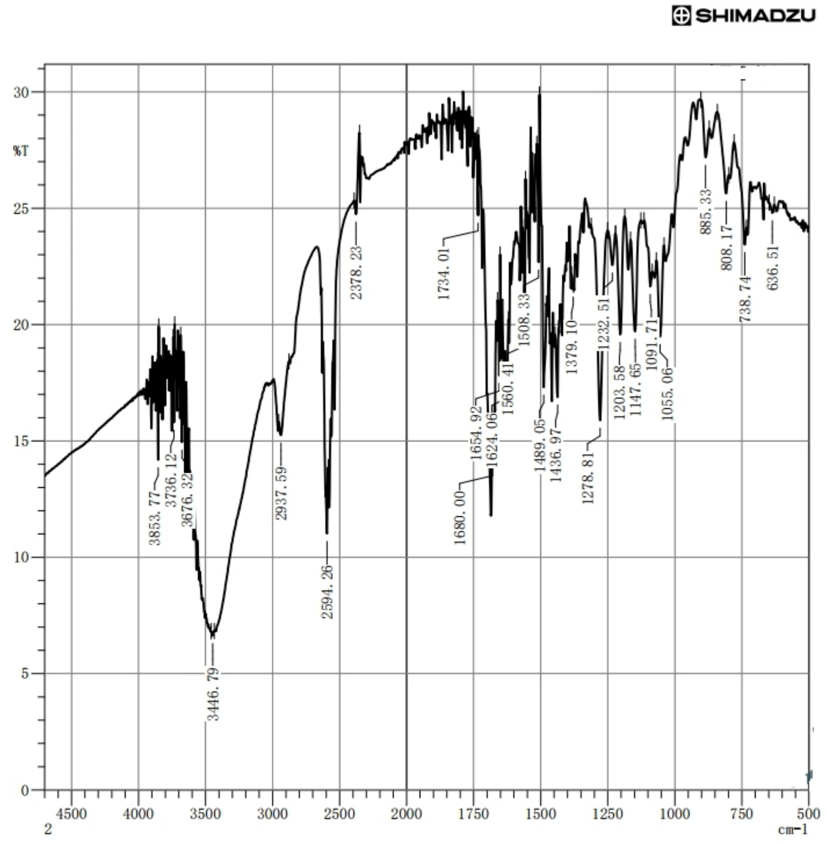

Figure S-2. ^{11}B NMR {^{1}H-decoupled} of **3** in DMSO-d$_6$.

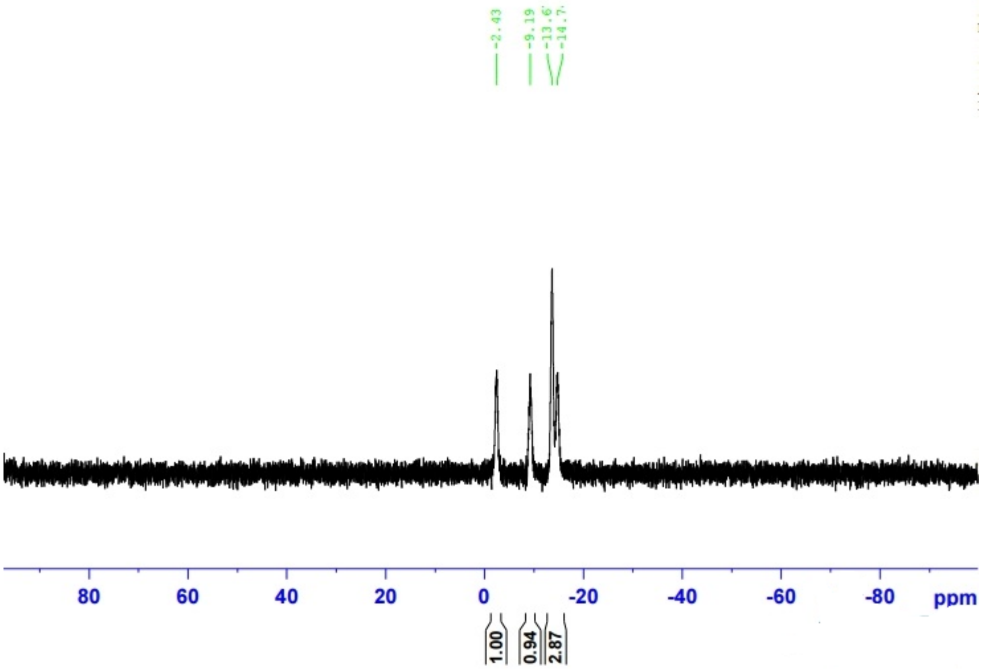

Figure S-3. ^{13}C NMR of **3** in DMSO-d$_6$.

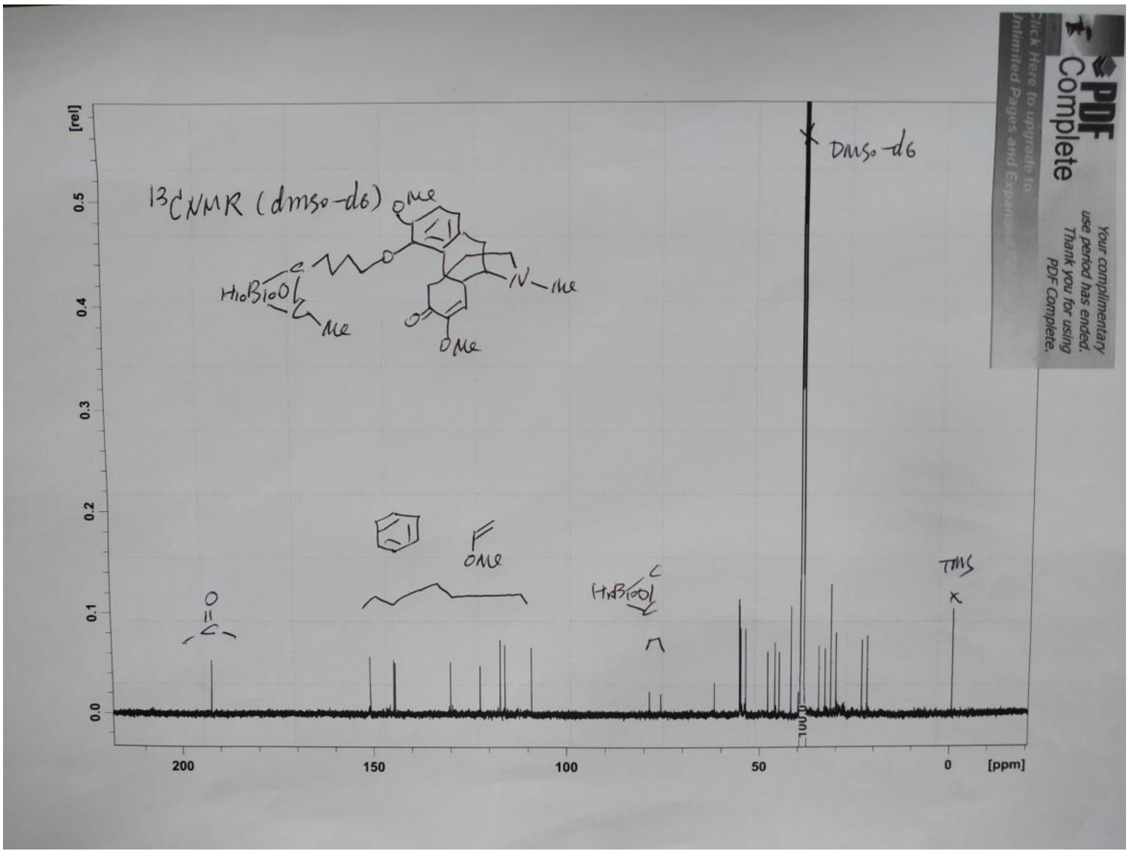

Figure S-4. ^{1}H NMR of **3** in DMSO-d_6.

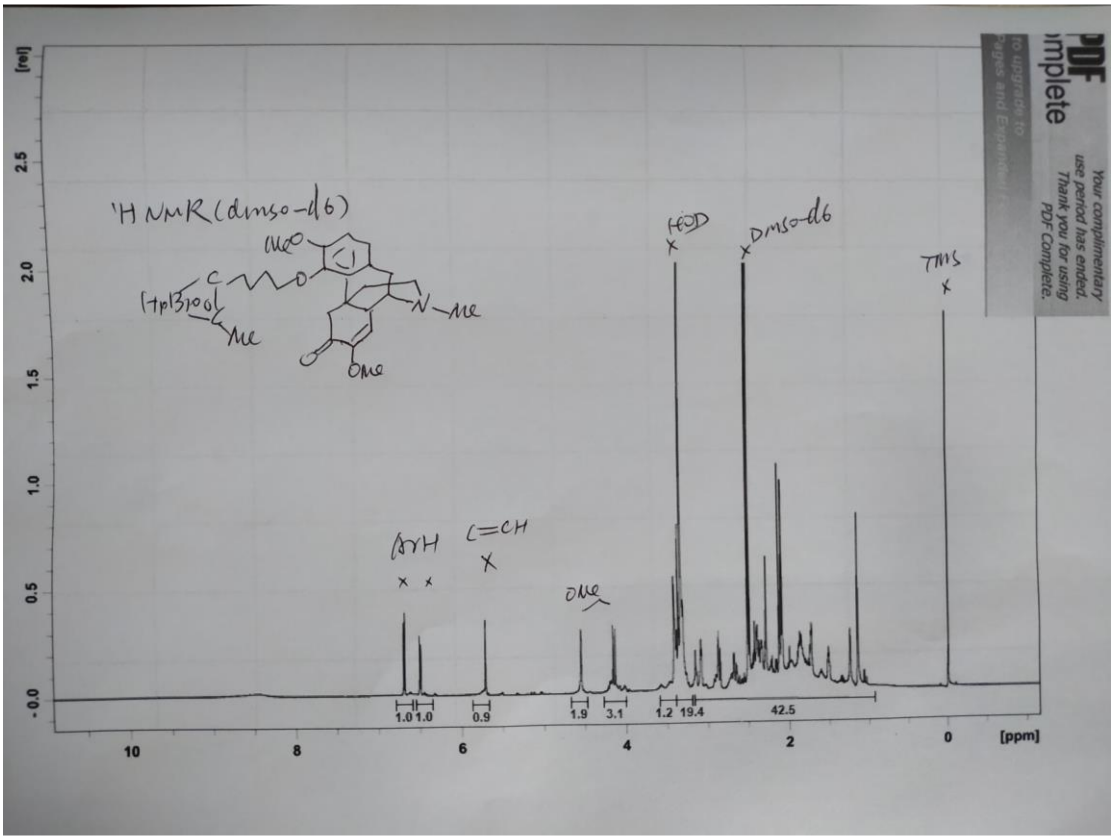

2. Cytotoxicity study. For IC_{50} analysis, the standard MTT method was used to analyze IC_{50} of compound 3. The IC_{50} determinations were performed in the presence of serial dilutions of inhibitor (1000 μ*M* to 3.906 μ*M*, 1% DMSO, final) in complete reaction buffer and incubated for 96 hours.

Figure S-5. IC_{50} dose responsive curve of **3** for rat FLS cells.

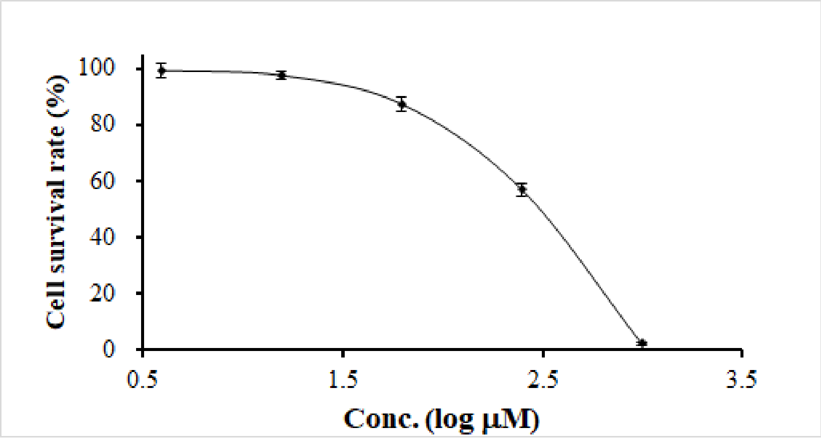

3. Molecular docking calculation. The molecular operating environment (MOE) software (Chemical Computing Group 2016) and protein-small molecule docking study Patch dock open source server (http://bioinfo3d.cs.tau.ac.il/PatchDock/) [2-5], which can predict favorable protein-ligand complex structures with high accuracy have been used to calculate the molecular interactions between ligand (small molecule) and receptor (protein). The three-dimensional (3D) crystal structures of MMP1 and MMP13 proteins were retrieved from the Protein Data Bank (PDB) (www.rcsb.org). Solvent impurities and co-crystallized ligand molecule and all water molecules were manually removed by Pymol 2.4 Software package, and then saved the proteins as .pdb files after adding Polar hydrogen atoms to the proteins. Both PDB files were uploaded into the Patch dock server for protein- small molecule docking simulation. Docking was performed with complex type configuration settings. PatchDock server follows a geometry-based molecular docking algorithm to find the docking transformations with good molecular shape complementarity. PatchDock algorithm separates the Connolly dot surface representation of the molecules into concave, convex and flat patches. These divided complementary patches are matched in order to generate candidate transformations and evaluated by geometric fit and atomic desolvation energy scoring functions. The results showed binding sites for **3**. The association details of each binding site are presented using the "ligand interactions" module of MOE. The calculated results of geometric shape complementarity score are listed at Table S-1. The docking models of **3** with varying MMPs are shown in Figure 1 and 2.

Table S-1. Geometric shape complementarity scores of sinomenine (1), methyl-*ortho*-carborane (2) and 3 (Å^2).

MMPs	MMP-1 collagenases			MMP-13 collagenases									
No	1	2	3	1	2	3	4	5	6	7	8	9	10
Collagenases	1FBL	1SU3	3SHI	4JPA	3ZXH	5B5O	1XUC	1XUD	1XUR	2OW9	2YIG	3ELM	3I7G
1	3970	4768	4632	4714	4786	4610	4744	4744	4816	4864	4648	4526	5138
2	2242	2388	2446	2228	2196	2300	2302	2292	2296	2262	2226	2348	2322
3	4536	5264	4642	5280	5084	5204	5312	5226	5308	5424	5116	5162	5172

MMPs	MMP-13 collagenases												
No	11	12	13	14	15	16	17	18	19	20	21	22	23
Collagenases	3KEK	3KRY	3OZX	3WV1	3WV3	4L19	5B5P	5BOT	5BPA	5UWM	6HV2	830C	1CXV
1	4860	4850	4858	4824	4648	4794	4682	4868	4840	4964	4474	4766	4618
2	2320	2276	2280	2278	2324	2242	2222	2314	2252	2294	2344	2232	2192
3	5522	5814	5582	5344	5380	5230	4990	5218	5572	5532	4730	4856	5280

MMPs	MMP-13 collagenases												
No	24	25	26	27	28	29	30	31	32	33	34	35	36
Collagenases	1ZTQ	2D1N	2OZR	3I7I	3KEC	3KEJ	3LJZ	3TVC	3WV2	4A7B	4JPA	5BOY	456C
1	4912	4826	5056	4884	4856	4792	4904	4378	4718	4528	4636	4760	4964
2	2328	2264	2394	2252	2272	2254	2360	2362	2286	2260	2158	2302	2252
3	5634	4904	5630	5114	5704	5434	5212	4598	5178	5188	5100	5228	5290

MMPs	MMP-13 collagenases												
No	37	38	39	40	41	42	43	44	45	46	47	48	49
Collagenases	2E2D	4FVL	5UWL	5UWN	1FML	4FU4	5UWK	4G0D	1EUB	1FLS	2PJT	1YOU	1PEX
1	4676	4456	4844	5160	4660	4480	4884	4702	4212	4162	4706	4706	4542
2	2326	2552	2220	2526	2164	2516	2288	2554	2160	2228	2172	2172	2240
3	5386	5206	5398	5552	4844	4964	5436	5724	4736	4546	5010	5010	4918

*3.1 Molecular docking results of **3** with MMP1-1FBL, 1SU3 and 3SHI.*

PATCHDOCK

Molecular Docking Algorithm Based on Shape Complementarity Principles

[About PatchDock] [Web Server] [Download] [Help] [FAQ] [References]

Receptor	Ligand	Complex Type	Clustering RMSD	User e-mail
1fbl1.pdb	4.pdb	drug	1.5	lzy930313@gmail.com

Solution No	Score	Area	ACE	Transformation
1	4536	546.10	-56.50	-1.50 1.13 -2.11 97.12 105.48 -4.03
2	4378	561.30	-120.70	-2.41 0.99 1.87 104.58 107.25 -1.35
3	4328	559.40	-88.43	1.91 -1.31 -0.75 99.51 102.59 27.64

Molecular Docking Algorithm Based on Shape Complementarity Principles

[About PatchDock] [Web Server] [Download] [Help] [FAQ] [References]

Receptor	Ligand	Complex Type	Clustering RMSD	User e-mail
3shi1.pdb	4.pdb	drug	1.5	1909853wct30001@student.must.edu.mo

Solution No	Score	Area	ACE	Transformation
1	4642	545.50	-101.63	-0.83 -1.10 0.97 22.26 -31.07 13.92
2	4578	562.10	-88.06	1.69 -1.42 -1.10 5.21 -23.53 -1.70
3	4540	605.80	-154.19	-2.83 -1.19 -2.54 3.35 -6.27 -3.69

Molecular Docking Algorithm Based on Shape Complementarity Principles

[About PatchDock] [Web Server] [Download] [Help] [FAQ] [References]

Receptor	Ligand	Complex Type	Clustering RMSD	User e-mail
1su31.pdb	4.pdb	drug	1.5	1909853wct30001@student.must.edu.mo

Solution No	Score	Area	ACE	Transformation
1	5264	600.10	-180.26	0.29 1.07 -0.76 15.54 -68.32 15.99
2	5258	578.40	-203.44	-2.43 -1.30 -1.53 48.43 -6.08 61.25
3	5234	617.30	-313.56	1.33 -0.39 2.59 21.16 -85.68 38.64
[illegible]	[illegible]	[illegible]	[illegible]	[illegible]

*3.2 Molecular docking results of **3** with MMP13-2YIG and other collageneses.*

PATCHDOCK

Molecular Docking Algorithm Based on Shape Complementarity Principles

[About PatchDock] [Web Server] [Download] [Help] [FAQ] [References]

Receptor	Ligand	Complex Type	Clustering RMSD	User e-mail
2yig1.pdb	4.pdb	drug	1.5	lzy930313@gmail.com

Solution No	Score	Area	ACE	Transformation
1	5116	544.70	-247.85	0.40 -0.68 0.67 32.35 7.57 31.40
2	5056	641.90	-319.05	2.39 0.15 -0.15 46.73 -22.26 25.97
3	5040	565.60	-248.66	0.08 -0.43 -0.08 32.17 14.54 27.18

Molecular Docking Algorithm Based on Shape Complementarity Principles

[About PatchDock] [Web Server] [Download] [Help] [FAQ] [References]

Receptor	Ligand	Complex Type	Clustering RMSD	User e-mail
1eub1.pdb	4.pdb	drug	1.5	1909853wct30001@student.must.edu.mo

Solution No	Score	Area	ACE	Transformation
1	4736	635.20	-324.04	-0.10 0.08 -1.65 13.22 10.59 -3.67
2	4704	623.50	-345.25	-2.41 -0.69 -1.89 1.33 14.22 3.39
3	4562	650.80	-346.55	-2.50 -1.01 -2.09 1.98 12.56 6.89

Molecular Docking Algorithm Based on Shape Complementarity Principles
[About PatchDock] [Web Server] [Download] [Help] [FAQ] [References]

Receptor	Ligand	Complex Type	Clustering RMSD	User e-mail
1fls1.pdb	4.pdb	drug	1.5	1909853wct30001@student.must.edu.mo

Solution No	Score	Area	ACE	Transformation
1	4546	591.20	-378.60	-2.15 0.33 -0.61 -6.53 -3.27 -11.81
2	4408	541.30	-271.63	0.76 1.25 -2.08 14.34 0.28 -10.68
3	4338	532.20	-327.88	-0.88 -0.87 0.63 -10.81 -8.38 5.27
[illegible]	[illegible]	[illegible]	[illegible]	[illegible]

Molecular Docking Algorithm Based on Shape Complementarity Principles
[About PatchDock] [Web Server] [Download] [Help] [FAQ] [References]

Receptor	Ligand	Complex Type	Clustering RMSD	User e-mail
1pex1.pdb	4.pdb	drug	1.5	1909853wct30001@student.must.edu.mo

Solution No	Score	Area	ACE	Transformation
1	4918	620.10	-69.51	-1.26 1.18 1.34 24.37 10.77 55.49
2	4780	534.60	-94.30	0.89 0.52 2.01 19.23 -16.70 59.81
3	4778	507.80	-50.54	-1.04 -0.34 -2.15 29.26 8.30 57.64

Molecular Docking Algorithm Based on Shape Complementarity Principles
[About PatchDock] [Web Server] [Download] [Help] [FAQ] [References]

Receptor	Ligand	Complex Type	Clustering RMSD	User e-mail
1xuc1.pdb	4.pdb	drug	1.5	1909853wct30001@student.must.edu.mo

Solution No	Score	Area	ACE	Transformation
1	5312	716.70	-408.70	1.93 0.68 -0.02 15.88 -6.77 57.90
2	5304	586.30	-270.35	0.80 -0.47 1.08 3.72 14.68 67.83
3	5256	577.90	-244.16	-1.16 -0.16 0.60 -1.25 14.15 50.86

Molecular Docking Algorithm Based on Shape Complementarity Principles
[About PatchDock] [Web Server] [Download] [Help] [FAQ] [References]

Receptor	Ligand	Complex Type	Clustering RMSD	User e-mail
1xud1.pdb	4.pdb	drug	1.5	1909853wct30001@student.must.edu.mo

Solution No	Score	Area	ACE	Transformation
1	5226	585.80	-280.28	0.80 -0.36 0.80 1.74 16.26 67.48
2	5108	574.20	-244.51	0.63 -0.76 0.49 4.20 23.14 69.71
3	5076	587.30	-257.38	0.33 0.33 -2.28 27.74 23.90 55.32

Molecular Docking Algorithm Based on Shape Complementarity Principles
[About PatchDock] [Web Server] [Download] [Help] [FAQ] [References]

Receptor	Ligand	Complex Type	Clustering RMSD	User e-mail
4jp41.pdb	4.pdb	drug	1.5	1909853wct30001@student.must.edu.mo

Solution No	Score	Area	ACE	Transformation
1	5280	700.80	-320.72	-2.78 -0.95 -1.18 57.48 -11.12 37.93
2	5232	735.90	-346.50	0.96 -1.00 -0.31 69.01 -10.85 42.87
3	5172	552.40	-250.93	0.62 -0.81 0.97 35.82 5.32 33.61

Molecular Docking Algorithm Based on Shape Complementarity Principles
[About PatchDock] [Web Server] [Download] [Help] [FAQ] [References]

Receptor	Ligand	Complex Type	Clustering RMSD	User e-mail
4fvl1.pdb	4.pdb	drug	1.5	1909853wct30001@student.must.edu.mo

Solution No	Score	Area	ACE	Transformation
1	5206	656.90	-139.06	2.56 -0.52 3.10 -2.15 37.83 7.99
2	5090	678.30	-275.86	3.00 0.22 0.05 -19.32 20.63 -3.24
3	5074	644.60	-111.97	3.01 0.76 0.10 -20.85 20.45 -11.75

Molecular Docking Algorithm Based on Shape Complementarity Principles

[About PatchDock] [Web Server] [Download] [Help] [FAQ] [References]

Receptor	Ligand	Complex Type	Clustering RMSD	User e-mail
4g0d1.pdb	4.pdb	drug	1.5	1909853wct30001@student.must.edu.mo

Solution No	Score	Area	ACE	Transformation
1	5724	656.10	117.08	0.85 -0.19 3.01 59.55 21.85 88.50
2	5514	656.20	10.03	-1.96 -0.86 2.05 33.15 14.36 36.12
3	5496	609.20	125.71	1.47 0.29 -0.07 34.09 31.33 84.36

Molecular Docking Algorithm Based on Shape Complementarity Principles

[About PatchDock] [Web Server] [Download] [Help] [FAQ] [References]

Receptor	Ligand	Complex Type	Clustering RMSD	User e-mail
1you1.pdb	4.pdb	drug	1.5	1909853wct30001@student.must.edu.mo

Solution No	Score	Area	ACE	Transformation
1	5010	623.90	-288.07	2.49 0.14 -1.39 43.05 52.75 13.69
2	4938	554.40	-111.34	2.44 1.18 1.36 51.19 29.57 -7.33
3	4934	583.30	-219.41	1.51 -0.45 -1.02 48.54 48.15 23.92

Molecular Docking Algorithm Based on Shape Complementarity Principles

[About PatchDock] [Web Server] [Download] [Help] [FAQ] [References]

Receptor	Ligand	Complex Type	Clustering RMSD	User e-mail
3i7g1.pdb	4.pdb	drug	1.5	1909853wct30001@student.must.edu.mo

Solution No	Score	Area	ACE	Transformation
1	5172	568.90	-263.13	0.66 -0.72 0.77 2.76 19.87 68.99
2	5074	547.80	-251.37	0.74 -0.40 1.11 2.35 14.14 66.96
3	5022	565.60	-213.07	0.04 -0.81 -0.17 3.78 29.03 67.21

Molecular Docking Algorithm Based on Shape Complementarity Principles

[About PatchDock] [Web Server] [Download] [Help] [FAQ] [References]

Receptor	Ligand	Complex Type	Clustering RMSD	User e-mail
1xur1.pdb	4.pdb	drug	1.5	1909853wct30001@student.must.edu.mo

Solution No	Score	Area	ACE	Transformation
1	5308	727.00	-422.54	1.90 0.47 0.03 15.56 -7.27 61.43
2	5166	561.40	-252.25	1.08 -0.41 1.29 6.46 12.75 68.86
3	5052	664.80	-312.74	1.54 -0.04 -3.11 -3.50 28.83 52.32

Molecular Docking Algorithm Based on Shape Complementarity Principles

[About PatchDock] [Web Server] [Download] [Help] [FAQ] [References]

Receptor	Ligand	Complex Type	Clustering RMSD	User e-mail
1cxv1.pdb	4.pdb	drug	1.5	1909853wct30001@student.must.edu.mo

Solution No	Score	Area	ACE	Transformation
1	5280	650.40	-228.95	3.00 -1.28 1.26 45.11 6.22 72.86
2	5132	631.60	-154.72	2.98 -1.09 -2.96 41.08 21.01 71.89
3	5106	581.30	-130.51	2.68 0.99 0.27 29.04 2.11 50.42

Molecular Docking Algorithm Based on Shape Complementarity Principles

[About PatchDock] [Web Server] [Download] [Help] [FAQ] [References]

Receptor	Ligand	Complex Type	Clustering RMSD	User e-mail
2ow91.pdb	4.pdb	drug	1.5	1909853wct30001@student.must.edu.mo

Solution No	Score	Area	ACE	Transformation
1	5424	744.10	-440.77	1.86 0.60 -0.07 78.34 -25.31 24.50
2	5400	752.40	-406.12	2.18 -0.42 -2.67 58.11 20.52 21.64
3	5366	731.70	-399.06	2.02 0.64 1.12 49.29 -2.17 8.55

Molecular Docking Algorithm Based on Shape Complementarity Principles

[About PatchDock] [Web Server] [Download] [Help] [FAQ] [References]

Receptor	Ligand	Complex Type	Clustering RMSD	User e-mail
2e2d1.pdb	4.pdb	drug	1.5	1909853wct30001@student.must.edu.mo

Solution No	Score	Area	ACE	Transformation
1	5386	623.40	-298.94	-2.77 0.85 1.38 -1.86 58.51 -7.23
2	5376	631.00	-311.64	-0.84 -0.91 -1.09 -9.58 80.73 11.24
3	5340	660.20	-143.44	2.57 -1.55 -0.26 0.90 62.17 4.92
[illegible]	[illegible]	[illegible]	[illegible]	[illegible]

Molecular Docking Algorithm Based on Shape Complementarity Principles

[About PatchDock] [Web Server] [Download] [Help] [FAQ] [References]

Receptor	Ligand	Complex Type	Clustering RMSD	User e-mail
3elm1.pdb	4.pdb	drug	1.5	1909853wct30001@student.must.edu.mo

Solution No	Score	Area	ACE	Transformation
1	5162	537.30	-240.46	0.76 -0.86 1.02 6.10 19.95 69.77
2	5014	545.90	-217.78	0.65 -0.64 0.90 3.07 17.76 68.98
3	4866	564.80	-300.99	0.80 -0.40 0.91 3.16 14.69 66.93
[illegible]	[illegible]	[illegible]	[illegible]	[illegible]

Molecular Docking Algorithm Based on Shape Complementarity Principles

[About PatchDock] [Web Server] [Download] [Help] [FAQ] [References]

Receptor	Ligand	Complex Type	Clustering RMSD	User e-mail
2yig1.pdb	4.pdb	drug	1.5	1909853wct30001@student.must.edu.mo

Solution No	Score	Area	ACE	Transformation
1	5116	544.70	-247.85	0.40 -0.68 0.67 32.35 7.57 31.40
2	5056	641.90	-319.05	2.39 0.15 -0.15 46.73 -22.26 25.97
3	5040	565.60	-248.66	0.08 -0.43 -0.08 32.17 14.54 27.18
4	5038	554.30	-205.81	0.13 -0.81 -0.19 35.76 15.41 31.69

Molecular Docking Algorithm Based on Shape Complementarity Principles

[About PatchDock] [Web Server] [Download] [Help] [FAQ] [References]

Receptor	Ligand	Complex Type	Clustering RMSD	User e-mail
3tvc1.pdb	4.pdb	drug	1.5	1909853wct30001@student.must.edu.mo

Solution No	Score	Area	ACE	Transformation
1	4598	633.80	-301.45	1.30 0.70 2.38 5.18 -22.28 -0.59
2	4588	565.40	-203.13	2.59 1.10 2.18 0.59 -18.51 -1.72
3	4540	540.40	-57.34	-0.03 -0.79 0.34 -12.12 -6.36 6.12
4	4458	561.80	-173.43	-1.15 0.27 1.16 -18.08 -21.72 -4.55

Molecular Docking Algorithm Based on Shape Complementarity Principles

[About PatchDock] [Web Server] [Download] [Help] [FAQ] [References]

Receptor	Ligand	Complex Type	Clustering RMSD	User e-mail
3i7i1.pdb	4.pdb	drug	1.5	1909853wct30001@student.must.edu.mo

Solution No	Score	Area	ACE	Transformation
1	5114	582.60	-257.55	2.50 -0.61 0.19 5.57 9.32 68.79
2	5098	538.40	-232.19	0.54 -0.82 0.46 3.60 23.78 68.87
3	4942	546.60	-238.22	2.75 -0.64 -1.27 0.96 23.05 68.13

Molecular Docking Algorithm Based on Shape Complementarity Principles

[About PatchDock] [Web Server] [Download] [Help] [FAQ] [References]

Receptor	Ligand	Complex Type	Clustering RMSD	User e-mail
2d1n1.pdb	4.pdb	drug	1.5	1909853wct30001@student.must.edu.mo

Solution No	Score	Area	ACE	Transformation
1	4704	673.40	-460.42	1.57 0.30 -0.94 -7.14 61.49 -27.50
2	4682	693.30	-464.62	-1.47 0.05 -1.24 -47.17 35.59 -21.51
3	4660	688.60	-481.59	-1.89 0.01 2.69 13.55 46.32 -39.55

Molecular Docking Algorithm Based on Shape Complementarity Principles
[About PatchDock] [Web Server] [Download] [Help] [FAQ] [References]

Receptor	Ligand	Complex Type	Clustering RMSD	User e-mail
3wv21.pdb	4.pdb	drug	1.5	1909853wct30001@student.must.edu.mo

Solution No	Score	Area	ACE	Transformation
1	5178	574.60	-139.02	-2.84 -0.04 -0.44 -36.88 -26.69 25.53
2	5112	598.10	-176.76	3.03 0.43 -1.96 -24.00 -8.15 23.29
3	5100	596.70	-178.82	2.00 0.20 0.60 -30.98 -33.74 31.86

Molecular Docking Algorithm Based on Shape Complementarity Principles
[About PatchDock] [Web Server] [Download] [Help] [FAQ] [References]

Receptor	Ligand	Complex Type	Clustering RMSD	User e-mail
3kej1.pdb	4.pdb	drug	1.5	1909853wct30001@student.must.edu.mo

Solution No	Score	Area	ACE	Transformation
1	5434	758.00	-390.60	0.15 -0.34 -1.69 54.25 32.75 26.40
2	5346	731.30	-315.80	0.18 -0.09 -1.61 52.25 32.32 23.74
3	5210	701.10	-301.49	0.04 0.18 0.50 67.95 29.83 17.85

Molecular Docking Algorithm Based on Shape Complementarity Principles
[About PatchDock] [Web Server] [Download] [Help] [FAQ] [References]

Receptor	Ligand	Complex Type	Clustering RMSD	User e-mail
6hv2.pdb	4.pdb	drug	1.5	1909853wct30001@student.must.edu.mo

Solution No	Score	Area	ACE	Transformation
1	4730	584.20	-217.98	2.48 -0.11 -2.14 22.96 4.38 34.99
2	4684	581.90	-262.87	-0.96 0.32 -2.24 31.80 -4.95 17.37
3	4600	516.50	-116.72	-1.38 0.72 -2.71 50.43 -16.15 3.56

Molecular Docking Algorithm Based on Shape Complementarity Principles
[About PatchDock] [Web Server] [Download] [Help] [FAQ] [References]

Receptor	Ligand	Complex Type	Clustering RMSD	User e-mail
830c1.pdb	4.pdb	drug	1.5	1909853wct30001@student.must.edu.mo

Solution No	Score	Area	ACE	Transformation
1	4856	532.20	-236.13	0.55 -0.64 1.14 3.11 15.52 68.11
2	4846	544.40	-256.35	0.51 -0.59 0.39 2.11 22.87 68.09
3	4836	722.90	-409.46	1.89 -0.05 -0.47 15.00 -0.51 69.22

Molecular Docking Algorithm Based on Shape Complementarity Principles
[About PatchDock] [Web Server] [Download] [Help] [FAQ] [References]

Receptor	Ligand	Complex Type	Clustering RMSD	User e-mail
5b5o1.pdb	4.pdb	drug	1.5	1909853wct30001@student.must.edu.mo

Solution No	Score	Area	ACE	Transformation
1	5204	739.10	-382.91	2.47 0.26 -0.23 -47.32 -20.73 11.40
2	5196	741.30	-363.46	-2.22 0.99 -2.82 -32.56 -10.62 -6.63
3	5180	579.70	-256.33	-2.51 0.33 2.57 4.61 4.69 7.12

Molecular Docking Algorithm Based on Shape Complementarity Principles
[About PatchDock] [Web Server] [Download] [Help] [FAQ] [References]

Receptor	Ligand	Complex Type	Clustering RMSD	User e-mail
4jpa1.pdb	4.pdb	drug	1.5	1909853wct30001@student.must.edu.mo

Solution No	Score	Area	ACE	Transformation
1	5100	558.20	-235.06	0.48 -0.71 -2.48 34.42 -6.79 32.25
2	4904	543.60	-236.18	0.68 -0.45 -2.10 34.45 0.41 30.90
3	4890	573.60	-291.56	2.13 -0.14 -2.91 34.67 2.93 29.97

Molecular Docking Algorithm Based on Shape Complementarity Principles

[About PatchDock] [Web Server] [Download] [Help] [FAQ] [References]

Receptor	Ligand	Complex Type	Clustering RMSD	User e-mail
4a7b1.pdb	4.pdb	drug	1.5	1909853wct30001@student.must.edu.mo

Solution No	Score	Area	ACE	Transformation
1	5188	710.60	-311.02	2.03 -0.44 -0.43 60.12 -16.18 39.93
2	4958	565.80	-180.16	-1.15 1.43 -1.66 47.77 -11.04 8.63
3	4934	529.90	-227.67	0.83 -0.75 0.96 36.78 4.78 34.48

Molecular Docking Algorithm Based on Shape Complementarity Principles

[About PatchDock] [Web Server] [Download] [Help] [FAQ] [References]

Receptor	Ligand	Complex Type	Clustering RMSD	User e-mail
5bot1.pdb	4.pdb	drug	1.5	1909853wct30001@student.must.edu.mo

Solution No	Score	Area	ACE	Transformation
1	5218	583.40	-313.69	0.74 -0.44 0.84 1.93 16.30 67.44
2	5116	577.70	-278.08	-1.11 -0.20 0.73 -0.92 12.29 51.34
3	5080	555.00	-262.68	-0.03 -0.68 -0.09 2.62 28.62 65.62
[illegible]	[illegible]	[illegible]	[illegible]	[illegible]

Molecular Docking Algorithm Based on Shape Complementarity Principles

[About PatchDock] [Web Server] [Download] [Help] [FAQ] [References]

Receptor	Ligand	Complex Type	Clustering RMSD	User e-mail
5bpa1.pdb	4.pdb	drug	1.5	1909853wct30001@student.must.edu.mo

Solution No	Score	Area	ACE	Transformation
1	5572	724.50	-378.46	-0.68 -0.15 -3.07 49.88 -12.99 58.52
2	5242	571.00	-231.91	0.34 -0.59 0.33 0.31 20.52 66.55
3	5226	703.30	-317.50	2.37 0.48 1.61 41.92 -22.14 63.71

Molecular Docking Algorithm Based on Shape Complementarity Principles

[About PatchDock] [Web Server] [Download] [Help] [FAQ] [References]

Receptor	Ligand	Complex Type	Clustering RMSD	User e-mail
4l191.pdb	4.pdb	drug	1.5	1909853wct30001@student.must.edu.mo

Solution No	Score	Area	ACE	Transformation
1	5230	614.60	-310.29	-1.15 -0.52 2.03 20.63 3.51 27.14
2	5216	644.10	-302.87	-0.79 0.33 -0.33 9.76 26.53 18.03
3	5172	631.50	-275.67	-1.09 0.79 -0.09 13.58 22.53 13.11

Molecular Docking Algorithm Based on Shape Complementarity Principles

[About PatchDock] [Web Server] [Download] [Help] [FAQ] [References]

Receptor	Ligand	Complex Type	Clustering RMSD	User e-mail
5uwk1.pdb	4.pdb	drug	1.5	1909853wct30001@student.must.edu.mo

Solution No	Score	Area	ACE	Transformation
1	5436	668.30	-320.23	0.31 1.09 1.10 -14.72 -5.30 18.53
2	5402	658.40	-284.27	-1.67 0.02 -1.02 -4.72 20.20 -7.81
3	5374	656.80	-231.68	-0.26 -0.82 1.55 -5.59 -0.40 8.40

Molecular Docking Algorithm Based on Shape Complementarity Principles

[About PatchDock] [Web Server] [Download] [Help] [FAQ] [References]

Receptor	Ligand	Complex Type	Clustering RMSD	User e-mail
3kek1.pdb	4.pdb	drug	1.5	1909853wct30001@student.must.edu.mo

Solution No	Score	Area	ACE	Transformation
1	5522	741.90	-389.79	0.11 -0.23 -1.68 54.57 33.38 24.79
2	5492	710.00	-385.96	-1.96 0.51 -1.23 38.47 23.94 7.39
3	5282	735.80	-401.53	-1.35 -0.26 1.86 47.70 7.50 16.75
[illegible]	[illegible]	[illegible]	[illegible]	[illegible]

Molecular Docking Algorithm Based on Shape Complementarity Principles

[About PatchDock] [Web Server] [Download] [Help] [FAQ] [References]

Receptor	Ligand	Complex Type	Clustering RMSD	User e-mail
5uwm1.pdb	4.pdb	drug	1.5	1909853wct30001@student.must.edu.mo

Solution No	Score	Area	ACE	Transformation
1	5532	697.50	-228.40	1.97 1.44 0.20 -61.07 132.54 -15.88
2	5398	682.30	-365.14	-1.59 -0.00 -0.61 -41.46 140.99 -11.66
3	5230	645.80	-189.06	0.67 0.76 1.38 -61.18 126.72 -8.22

Molecular Docking Algorithm Based on Shape Complementarity Principles

[About PatchDock] [Web Server] [Download] [Help] [FAQ] [References]

Receptor	Ligand	Complex Type	Clustering RMSD	User e-mail
5b5p1.pdb	4.pdb	drug	1.5	1909853wct30001@student.must.edu.mo

Solution No	Score	Area	ACE	Transformation
1	4990	575.30	-211.09	0.59 -0.52 0.43 -35.32 24.39 31.85
2	4982	568.50	-140.71	-0.72 1.01 -2.57 -16.99 8.15 9.78
3	4944	522.10	-222.19	0.83 -0.21 0.90 -34.99 17.55 29.97

Molecular Docking Algorithm Based on Shape Complementarity Principles

[About PatchDock] [Web Server] [Download] [Help] [FAQ] [References]

Receptor	Ligand	Complex Type	Clustering RMSD	User e-mail
5boy1.pdb	4.pdb	drug	1.5	1909853wct30001@student.must.edu.mo

Solution No	Score	Area	ACE	Transformation
1	5228	578.40	-290.07	0.57 -0.40 0.75 -0.11 16.41 66.49
2	5200	568.80	-210.22	-0.95 0.92 -2.92 14.15 13.72 43.11
3	5150	693.70	-301.39	-1.50 0.50 2.47 42.26 -8.57 51.30

Molecular Docking Algorithm Based on Shape Complementarity Principles

[About PatchDock] [Web Server] [Download] [Help] [FAQ] [References]

Receptor	Ligand	Complex Type	Clustering RMSD	User e-mail
3wv31.pdb	4.pdb	drug	1.5	1909853wct30001@student.must.edu.mo

Solution No	Score	Area	ACE	Transformation
1	5380	644.30	-281.67	-0.62 0.32 -0.67 44.42 8.62 -16.67
2	5290	589.90	-284.92	-2.29 0.36 2.42 34.07 -14.32 -31.17
3	5196	651.60	-291.66	-0.59 0.56 -0.44 43.29 7.10 -19.88
[illegible]	[illegible]	[illegible]	[illegible]	[illegible]

Molecular Docking Algorithm Based on Shape Complementarity Principles

[About PatchDock] [Web Server] [Download] [Help] [FAQ] [References]

Receptor	Ligand	Complex Type	Clustering RMSD	User e-mail
3wv11.pdb	4.pdb	drug	1.5	1909853wct30001@student.must.edu.mo

Solution No	Score	Area	ACE	Transformation
1	5344	622.80	-218.39	-1.40 -0.97 0.50 13.53 -19.35 -18.57
2	5242	637.40	-216.17	-0.94 -1.06 1.11 17.24 -22.92 -15.80
3	5204	604.40	-207.06	1.67 0.83 -0.93 17.15 -1.34 -28.51

Molecular Docking Algorithm Based on Shape Complementarity Principles

[About PatchDock] [Web Server] [Download] [Help] [FAQ] [References]

Receptor	Ligand	Complex Type	Clustering RMSD	User e-mail
3kec1.pdb	4.pdb	drug	1.5	1909853wct30001@student.must.edu.mo

Solution No	Score	Area	ACE	Transformation
1	5704	762.40	-413.30	0.20 -0.36 -0.50 78.00 23.92 43.60
2	5654	760.20	-419.45	-2.55 -0.01 2.70 56.31 24.80 -3.22
3	5252	729.80	-389.33	1.19 -0.52 2.52 42.19 17.68 13.97

Molecular Docking Algorithm Based on Shape Complementarity Principles
[About PatchDock] [Web Server] [Download] [Help] [FAQ] [References]

Receptor	Ligand	Complex Type	Clustering RMSD	User e-mail
3zxh1.pdb	4.pdb	drug	1.5	1909853wct30001@student.must.edu.mo

Solution No	Score	Area	ACE	Transformation
1	5084	535.00	-244.57	0.80 -0.84 0.96 6.07 20.43 69.32
2	5062	702.00	-306.48	-1.68 -0.38 -1.70 37.84 10.05 59.43
3	5020	706.90	-333.57	0.94 -1.11 0.20 37.39 2.62 77.47

Molecular Docking Algorithm Based on Shape Complementarity Principles
[About PatchDock] [Web Server] [Download] [Help] [FAQ] [References]

Receptor	Ligand	Complex Type	Clustering RMSD	User e-mail
456c1.pdb	4.pdb	drug	1.5	1909853wct30001@student.must.edu.mo

Solution No	Score	Area	ACE	Transformation
1	5290	712.00	-288.02	-2.30 1.00 -1.18 28.96 -6.42 46.73
2	5036	744.40	-473.95	-0.36 -0.47 0.19 11.08 1.67 61.93
3	4980	742.10	-397.33	1.83 -0.11 -0.40 16.42 -1.03 68.12

Molecular Docking Algorithm Based on Shape Complementarity Principles
[About PatchDock] [Web Server] [Download] [Help] [FAQ] [References]

Receptor	Ligand	Complex Type	Clustering RMSD	User e-mail
5uwl1.pdb	4.pdb	drug	1.5	1909853wct30001@student.must.edu.mo

Solution No	Score	Area	ACE	Transformation
1	5398	607.10	-146.37	1.23 -0.25 -2.80 72.94 -16.77 27.41
2	5394	606.40	-122.01	-2.37 0.23 3.01 73.37 -13.13 8.22
3	5390	599.60	-129.12	-2.08 0.36 2.90 71.37 -15.40 6.49
4	[illegible]	[illegible]	[illegible]	[illegible]

Molecular Docking Algorithm Based on Shape Complementarity Principles
[About PatchDock] [Web Server] [Download] [Help] [FAQ] [References]

Receptor	Ligand	Complex Type	Clustering RMSD	User e-mail
1ztq1.pdb	4.pdb	drug	1.5	1909853wct30001@student.must.edu.mo

Solution No	Score	Area	ACE	Transformation
1	5634	702.60	-45.69	2.54 0.37 -1.98 4.40 70.57 58.08
2	5600	707.60	-89.55	2.08 0.52 -1.71 2.56 69.89 58.37
3	5558	665.80	31.57	-1.56 -0.01 1.13 -6.14 38.41 43.71

Molecular Docking Algorithm Based on Shape Complementarity Principles
[About PatchDock] [Web Server] [Download] [Help] [FAQ] [References]

Receptor	Ligand	Complex Type	Clustering RMSD	User e-mail
3ljz1.pdb	4.pdb	drug	1.5	1909853wct30001@student.must.edu.mo

Solution No	Score	Area	ACE	Transformation
1	5212	647.70	-181.52	2.05 1.15 0.00 20.19 75.49 64.66
2	5088	633.30	-146.88	2.01 0.66 -0.05 17.46 76.09 70.92
3	5084	651.10	-125.11	1.81 1.34 -2.37 40.18 89.72 70.79

Molecular Docking Algorithm Based on Shape Complementarity Principles
[About PatchDock] [Web Server] [Download] [Help] [FAQ] [References]

Receptor	Ligand	Complex Type	Clustering RMSD	User e-mail
3o2x1.pdb	4.pdb	drug	1.5	1909853wct30001@student.must.edu.mo

Solution No	Score	Area	ACE	Transformation
1	5582	723.90	-183.33	1.13 -1.39 1.47 30.88 87.70 95.01
2	5398	724.70	-187.16	1.92 1.39 0.48 25.62 74.23 68.94
3	5384	736.70	-210.32	2.96 1.53 -0.16 31.08 73.62 67.03

Molecular Docking Algorithm Based on Shape Complementarity Principles
[About PatchDock] [Web Server] [Download] [Help] [FAQ] [References]

Receptor	Ligand	Complex Type	Clustering RMSD	User e-mail
3kry1.pdb	4.pdb	drug	1.5	1909853wct30001@student.must.edu.mo

Solution No	Score	Area	ACE	Transformation
1	5814	659.70	-129.10	-2.37 -1.04 -1.35 -12.89 51.34 42.72
2	5764	670.70	-66.55	-1.30 -1.17 2.39 3.90 31.20 41.62
3	5636	658.60	-171.73	-2.20 -0.98 -1.35 -11.72 53.08 42.84
[illegible]	[illegible]	[illegible]	[illegible]	[illegible]

Molecular Docking Algorithm Based on Shape Complementarity Principles
[About PatchDock] [Web Server] [Download] [Help] [FAQ] [References]

Receptor	Ligand	Complex Type	Clustering RMSD	User e-mail
2pjt1.pdb	4.pdb	drug	1.5	1909853wct30001@student.must.edu.mo

Solution No	Score	Area	ACE	Transformation
1	4856	594.80	-67.27	3.05 -0.00 -0.70 -4.54 32.79 71.90
2	4846	561.30	-52.57	2.94 -0.31 -0.59 -3.55 30.70 75.64
3	4808	540.50	-170.51	0.51 0.22 -0.71 1.23 34.99 99.71
[illegible]	[illegible]	[illegible]	[illegible]	[illegible]

Molecular Docking Algorithm Based on Shape Complementarity Principles
[About PatchDock] [Web Server] [Download] [Help] [FAQ] [References]

Receptor	Ligand	Complex Type	Clustering RMSD	User e-mail
5uwn1.pdb	4.pdb	drug	1.5	1909853wct30001@student.must.edu.mo

Solution No	Score	Area	ACE	Transformation
1	5552	754.40	-182.96	1.21 -0.04 0.71 3.90 -14.49 35.89
2	5424	618.70	-181.03	1.22 0.73 -3.11 21.61 -5.58 39.45
3	5378	743.80	-197.55	1.92 -0.03 0.50 5.41 -17.36 36.35
[illegible]	[illegible]	[illegible]	[illegible]	[illegible]

Molecular Docking Algorithm Based on Shape Complementarity Principles
[About PatchDock] [Web Server] [Download] [Help] [FAQ] [References]

Receptor	Ligand	Complex Type	Clustering RMSD	User e-mail
4fu41.pdb	4.pdb	drug	1.5	1909853wct30001@student.must.edu.mo

Solution No	Score	Area	ACE	Transformation
1	4964	577.70	-108.17	-0.13 -0.09 2.24 17.30 17.36 27.47
2	4936	592.00	-205.26	2.36 0.00 -1.62 9.96 47.55 33.70
3	4890	544.80	-125.88	2.55 0.23 2.36 30.78 27.71 29.52

Molecular Docking Algorithm Based on Shape Complementarity Principles
[About PatchDock] [Web Server] [Download] [Help] [FAQ] [References]

Receptor	Ligand	Complex Type	Clustering RMSD	User e-mail
2ozr1.pdb	4.pdb	drug	1.5	1909853wct30001@student.must.edu.mo

Solution No	Score	Area	ACE	Transformation
1	5630	780.40	27.32	2.08 -1.25 0.07 10.34 24.31 29.06
2	5628	755.70	-75.16	-0.78 -0.53 2.13 9.05 13.48 14.10
3	5554	649.50	-84.50	0.44 0.21 -0.67 27.34 -14.65 32.52

4. References

1. Zhu, Y.; Ang, T.P.; Carpenter, K.; Maguire, J.A.; Hosmane, N.S.; Takagaki, M. Substituted Carborane-Appended Water-Soluble Single-Wall Carbon Nanotubes: New Approach to Boron Neutron Capture Therapy Drug Delivery. *J. Am. Chem. Soc*. **2005**, *127*, 9875–9880.

2. Lang, P.T.; Brozell, S.R.; Mukherjee, S.; Pettersen, E.F.; Meng, E.C.; Thomas, V. et al., DOCK 6: combining techniques to model RNA-small molecule complexes. *RNA (New York, N.Y.)*, **2009**, *15*, 1219–1230.
3. Schneidman-Duhovny, D.; Inbar, Y.; Nussinov, R.; Wolfson, H.J. PatchDock and SymmDock: Servers for rigid and symmetric docking. *Nucleic Acids Res*. **2005**, *33*, W363–W367.
4. Salentin, S.; Schreiber, S.; Haupt, V.J.; Adasme, M.F.; Schroeder, M. PLIP: fully automated protein–ligand interaction profiler. *Nucl. Acids Res*. **2015**, *43(W1)*, W443–W447.
5. De Lano, W.L. The PyMOL Molecular Graphics System. San Carlos, CA, USA: De Lano Scientific, **2004**.
